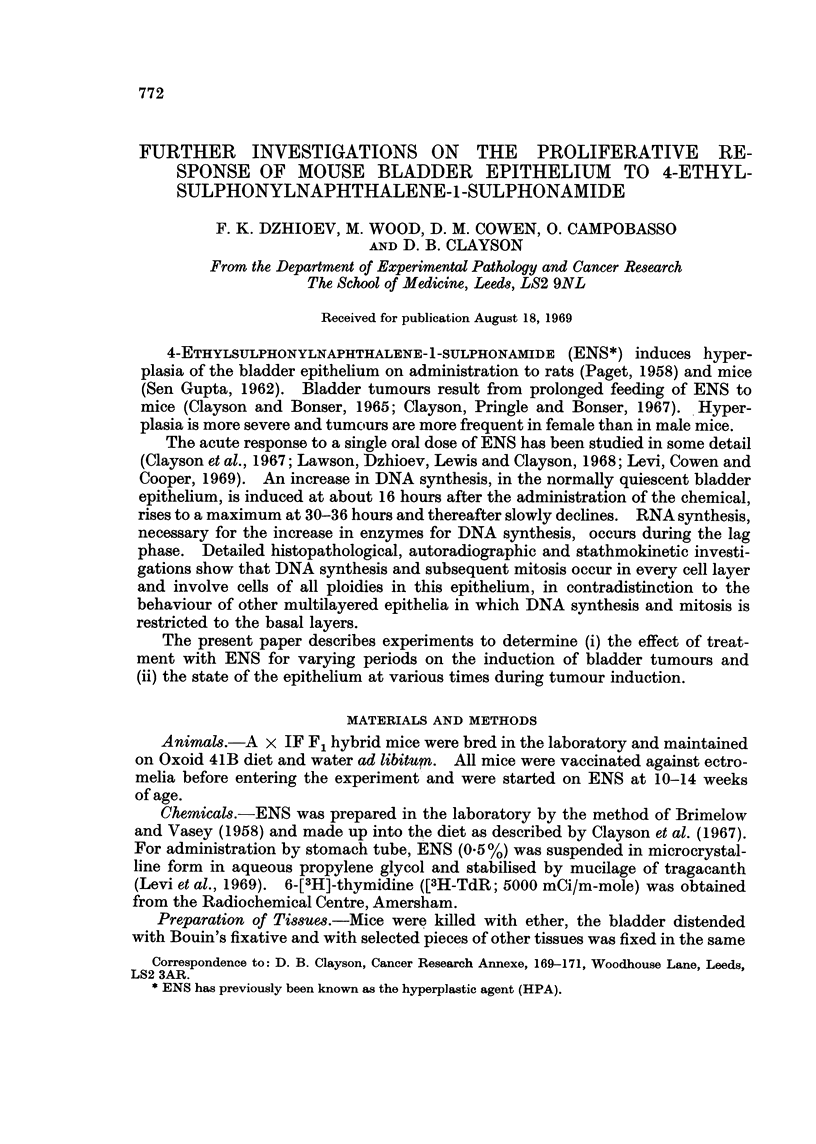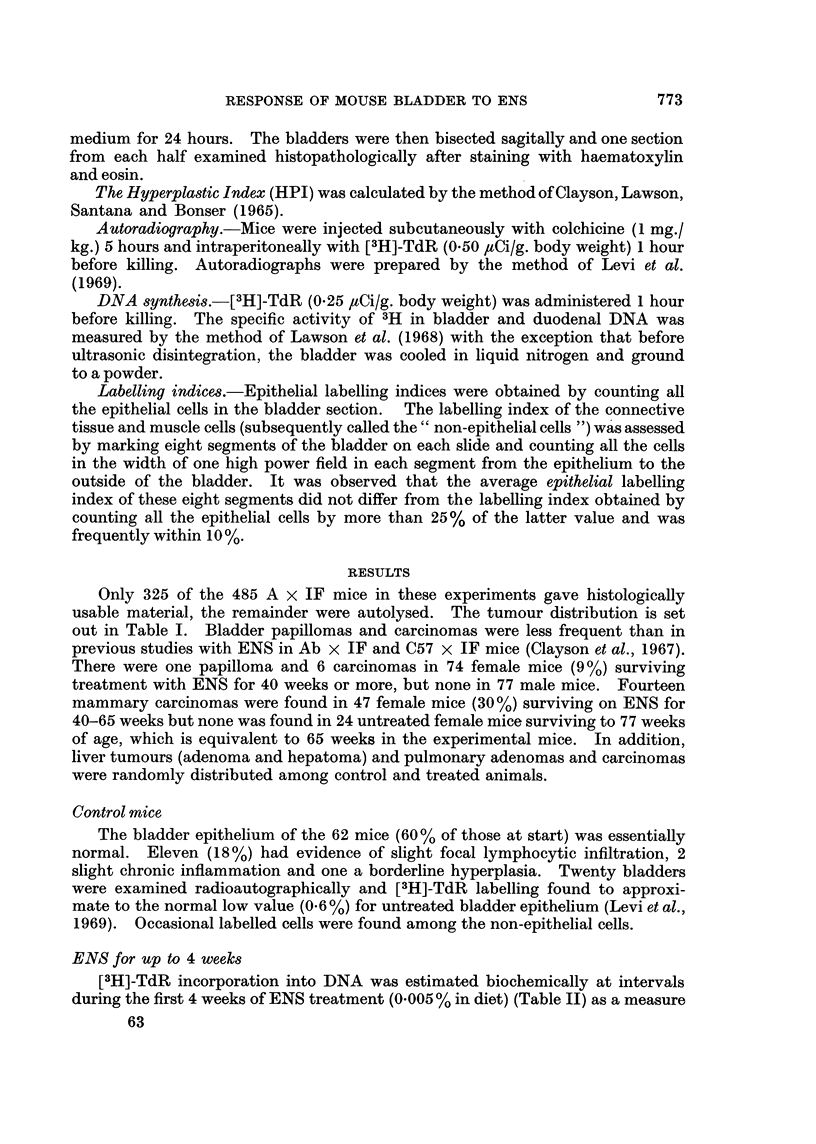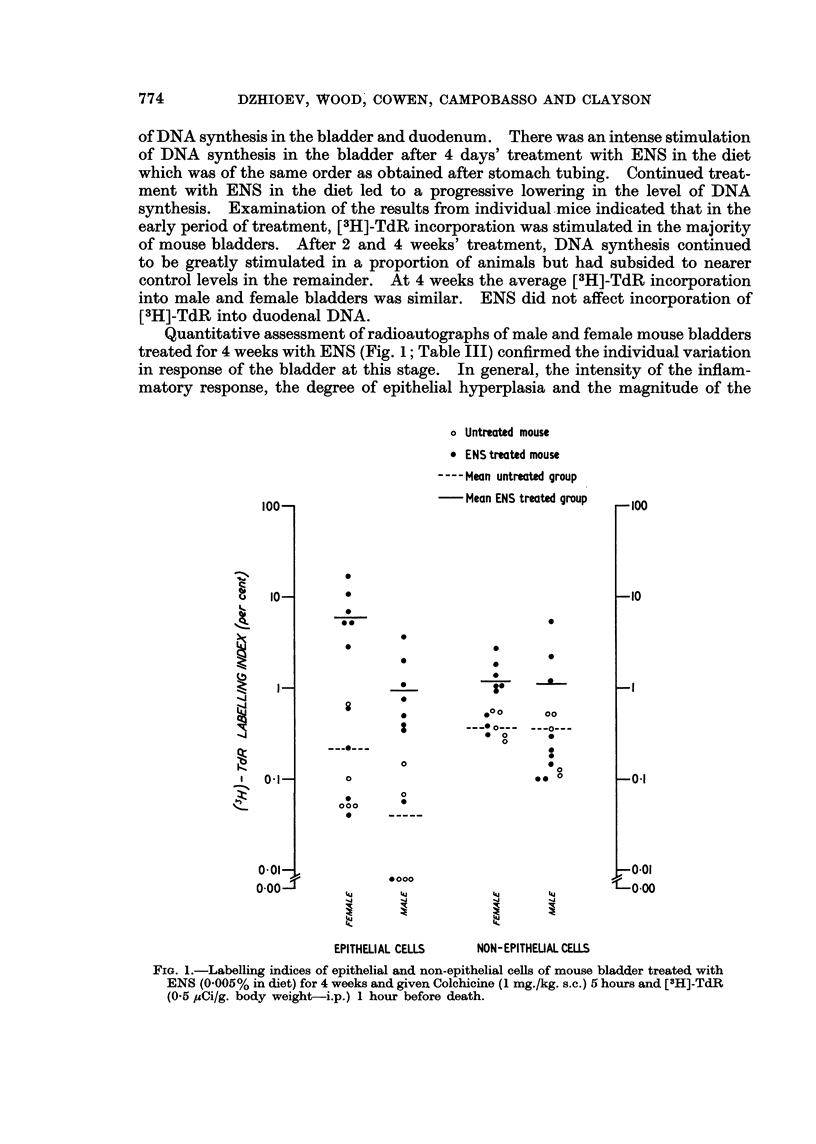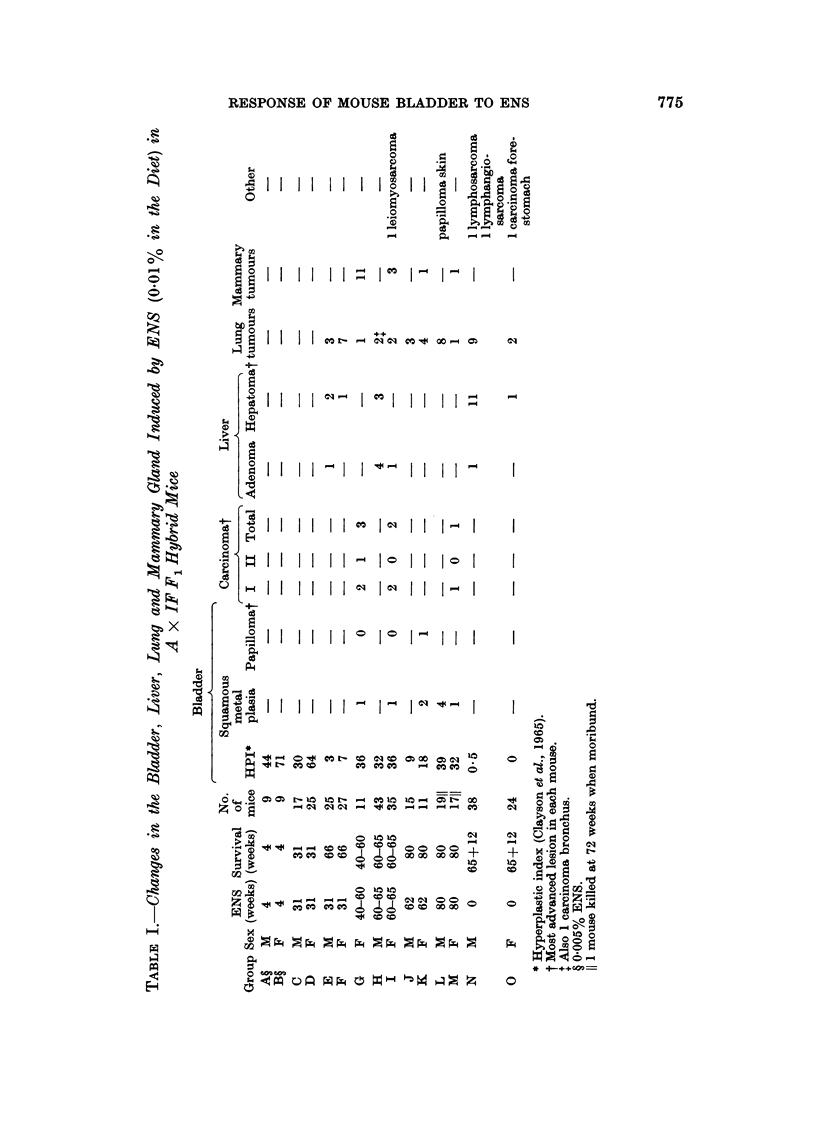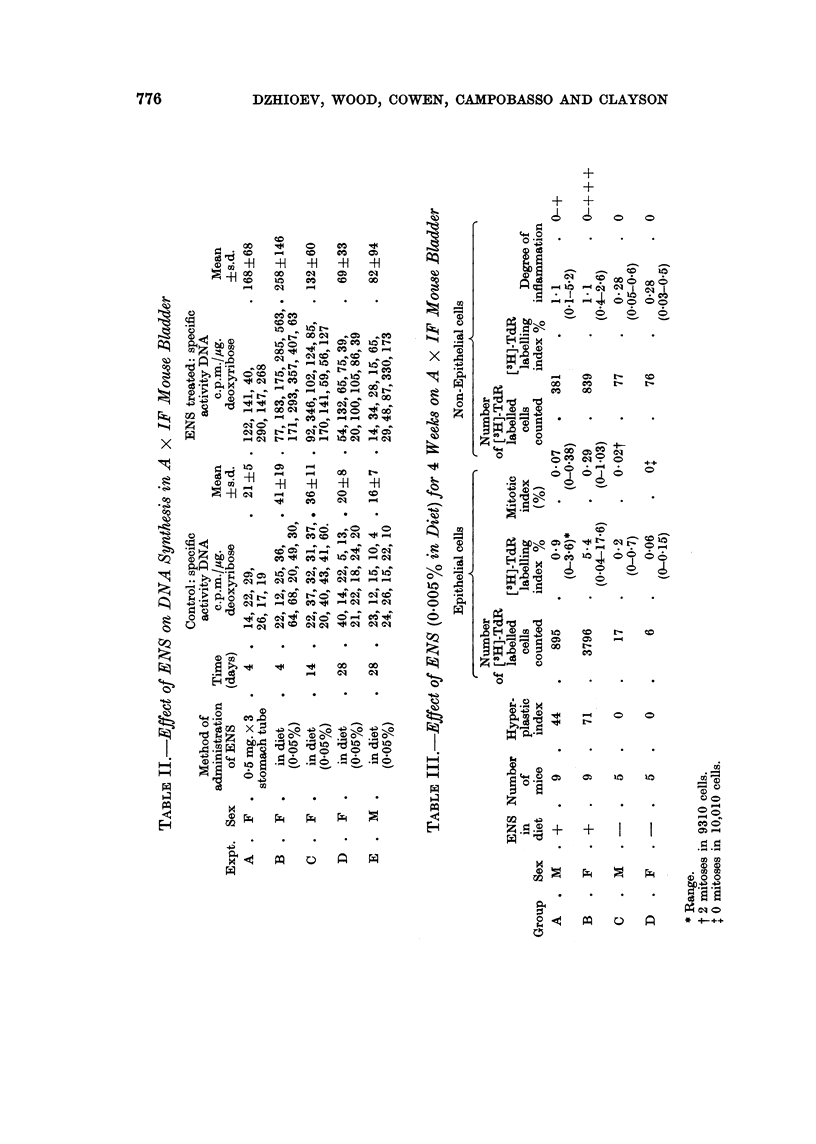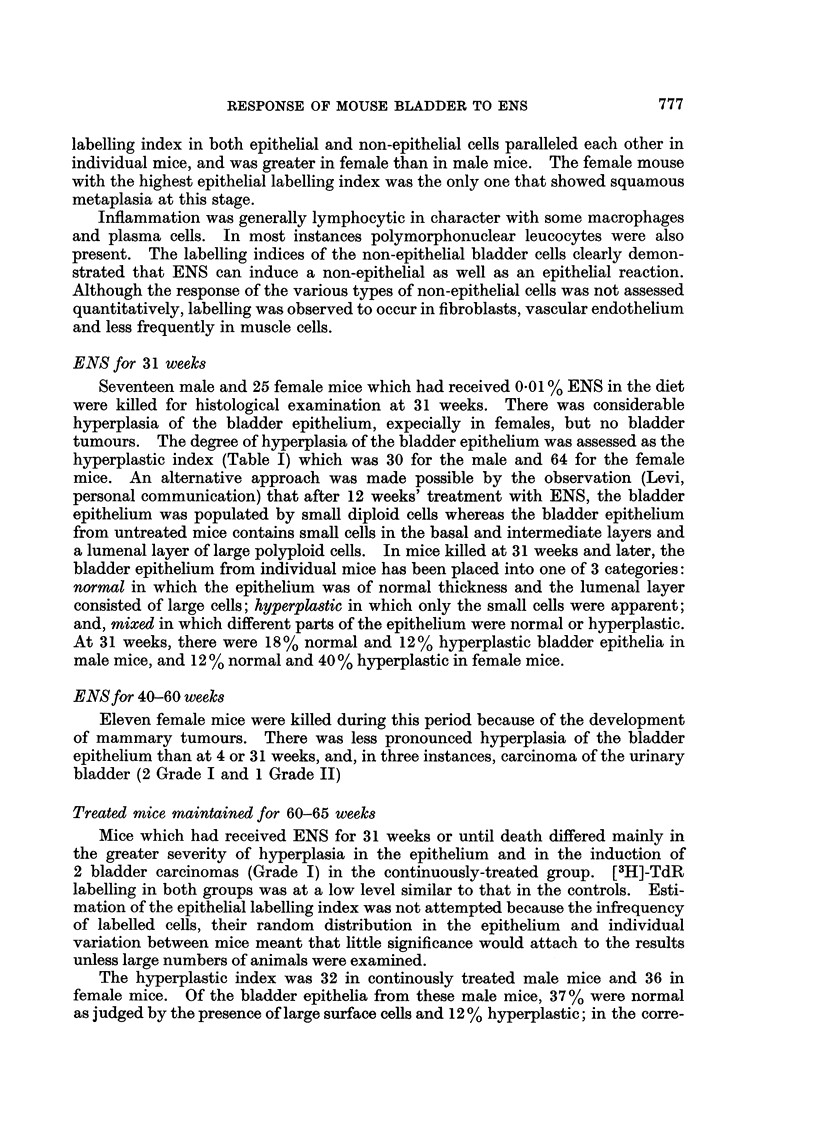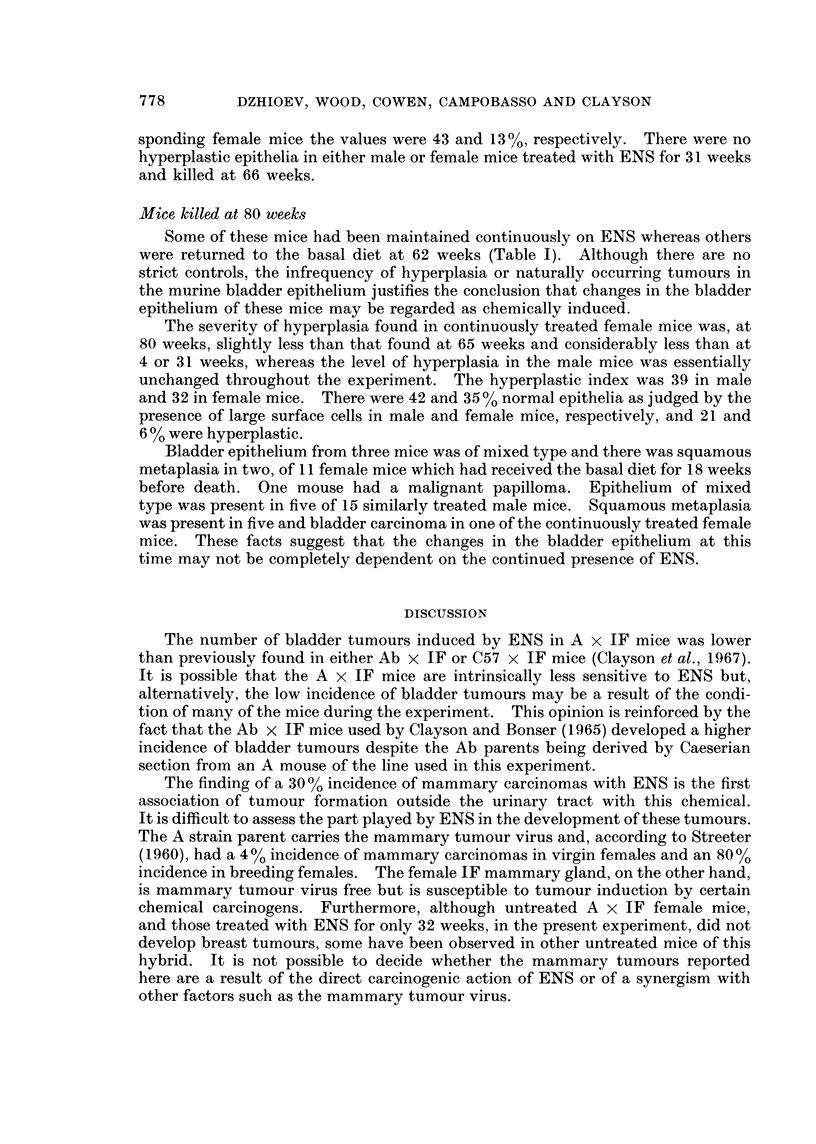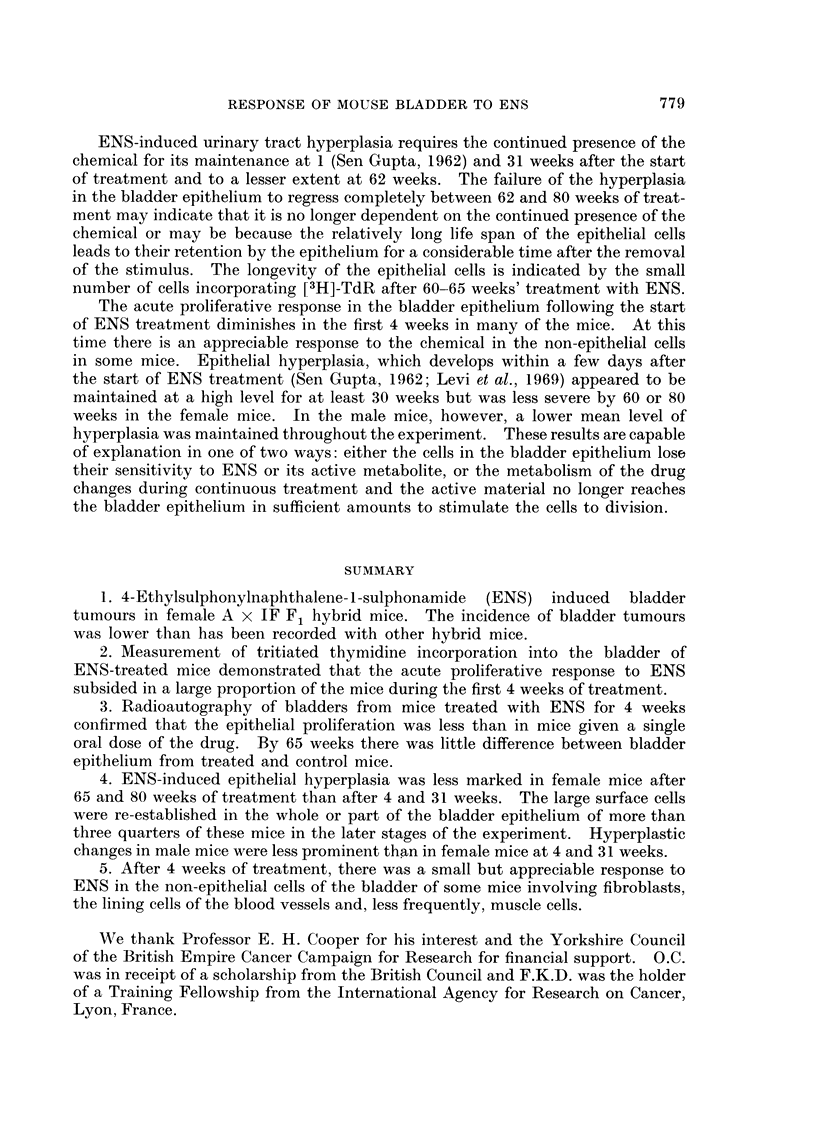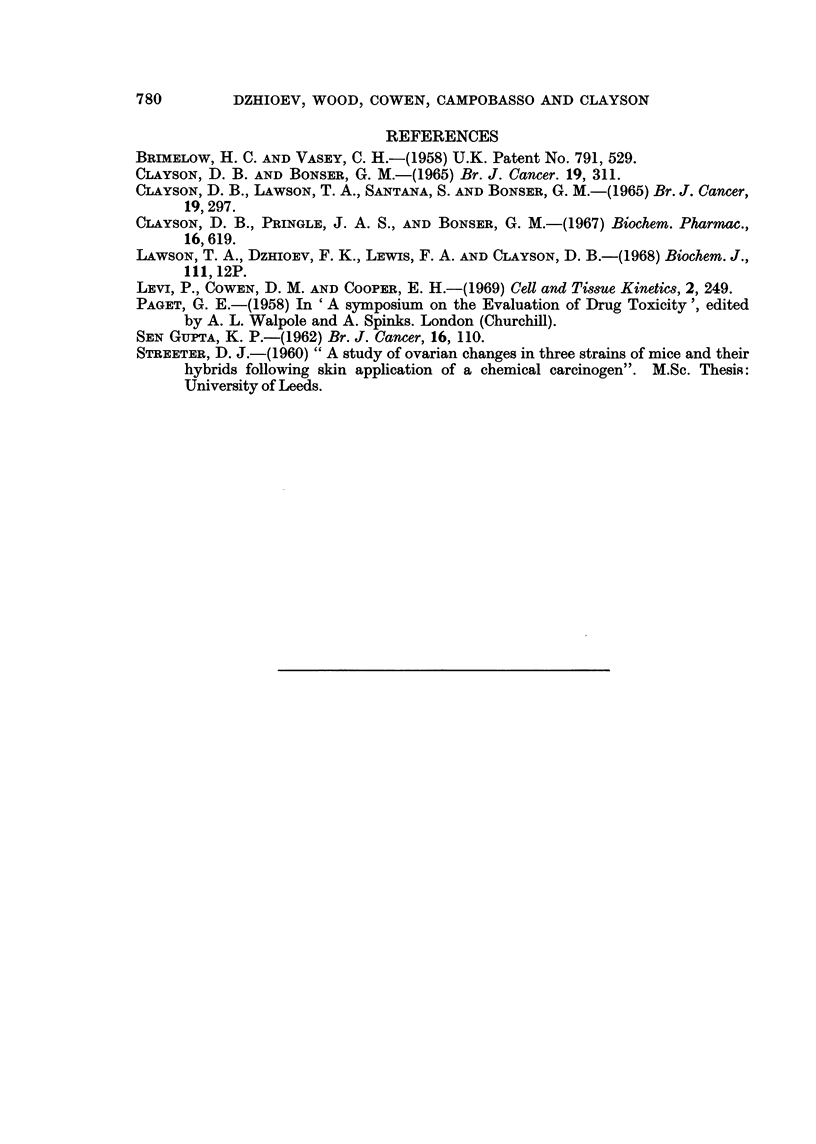# Further investigations on the proliferative response of mouse bladder epithelium to 4-ethyl-sulphonylnaphthalene-1-sulphonamide.

**DOI:** 10.1038/bjc.1969.96

**Published:** 1969-12

**Authors:** F. K. Dzhioev, M. Wood, D. M. Cowen, O. Campobasso, D. B. Clayson


### FURTHER INVESTIGATIONS ON THE PROLIFERATIVE RESPONSE OF MOUSE BLADDER EPITHELIUM TO 4-ETHYLSULPHONYLNAPHTHALENE-1-SULPHONAMIDE

F. K. DZHIOEV, M. WOOD, D. M. COWEN, O. CAMPOBASSO AND D. B. CLAYSON

*From the Department of Experimental Pathology and Cancer Research The School of Medicine, Leeds, LS2 9NL*

Received for publication August 18, 1969

4-ETHYLSULPHONYLNAPHTHALENE-1-SULPHONAMIDE (ENS*) induces hyperplasia of the bladder epithelium on administration to rats (Paget, 1958) and mice (Sen Gupta, 1962). Bladder tumours result from prolonged feeding of ENS to mice (Clayson and Bonser, 1965; Clayson, Pringle and Bonser, 1967). Hyperplasia is more severe and tumours are more frequent in female than in male mice.

The acute response to a single oral dose of ENS has been studied in some detail (Clayson *et al.*, 1967; Lawson, Dzhioev, Lewis and Clayson, 1968; Levi, Cowen and Cooper, 1969). An increase in DNA synthesis, in the normally quiescent bladder epithelium, is induced at about 16 hours after the administration of the chemical, rises to a maximum at 30–36 hours and thereafter slowly declines. RNA synthesis, necessary for the increase in enzymes for DNA synthesis, occurs during the lag phase. Detailed histopathological, autoradiographic and stathmokinetic investigations show that DNA synthesis and subsequent mitosis occur in every cell layer and involve cells of all ploidies in this epithelium, in contradistinction to the behaviour of other multilayered epithelia in which DNA synthesis and mitosis is restricted to the basal layers.

The present paper describes experiments to determine (i) the effect of treatment with ENS for varying periods on the induction of bladder tumours and (ii) the state of the epithelium at various times during tumour induction.

### MATERIALS AND METHODS

*Animals.*—A × IF $F_1$ hybrid mice were bred in the laboratory and maintained on Oxoid 41B diet and water *ad libitum*. All mice were vaccinated against ectromelia before entering the experiment and were started on ENS at 10–14 weeks of age.

*Chemicals.*—ENS was prepared in the laboratory by the method of Brimelow and Vasey (1958) and made up into the diet as described by Clayson *et al.* (1967). For administration by stomach tube, ENS (0·5%) was suspended in microcrystalline form in aqueous propylene glycol and stabilised by mucilage of tragacanth (Levi *et al.*, 1969). 6-[$^3$H]-thymidine ([$^3$H-TdR; 5000 mCi/m-mole) was obtained from the Radiochemical Centre, Amersham.

*Preparation of Tissues.*—Mice were killed with ether, the bladder distended with Bouin's fixative and with selected pieces of other tissues was fixed in the same

Correspondence to: D. B. Clayson, Cancer Research Annexe, 169–171, Woodhouse Lane, Leeds, LS2 3AR.

* ENS has previously been known as the hyperplastic agent (HPA).

medium for 24 hours. The bladders were then bisected sagitally and one section from each half examined histopathologically after staining with haematoxylin and eosin.

*The Hyperplastic Index* (HPI) was calculated by the method of Clayson, Lawson, Santana and Bonser (1965).

*Autoradiography.*—Mice were injected subcutaneously with colchicine (1 mg./kg.) 5 hours and intraperitoneally with [$^3$H]-TdR (0·50 $\mu$Ci/g. body weight) 1 hour before killing. Autoradiographs were prepared by the method of Levi *et al.* (1969).

*DNA synthesis.*—[$^3$H]-TdR (0·25 $\mu$Ci/g. body weight) was administered 1 hour before killing. The specific activity of $^3$H in bladder and duodenal DNA was measured by the method of Lawson *et al.* (1968) with the exception that before ultrasonic disintegration, the bladder was cooled in liquid nitrogen and ground to a powder.

*Labelling indices.*—Epithelial labelling indices were obtained by counting all the epithelial cells in the bladder section. The labelling index of the connective tissue and muscle cells (subsequently called the " non-epithelial cells ") was assessed by marking eight segments of the bladder on each slide and counting all the cells in the width of one high power field in each segment from the epithelium to the outside of the bladder. It was observed that the average *epithelial* labelling index of these eight segments did not differ from the labelling index obtained by counting all the epithelial cells by more than 25% of the latter value and was frequently within 10%.

### RESULTS

Only 325 of the 485 A × IF mice in these experiments gave histologically usable material, the remainder were autolysed. The tumour distribution is set out in Table I. Bladder papillomas and carcinomas were less frequent than in previous studies with ENS in Ab × IF and C57 × IF mice (Clayson *et al.*, 1967). There were one papilloma and 6 carcinomas in 74 female mice (9%) surviving treatment with ENS for 40 weeks or more, but none in 77 male mice. Fourteen mammary carcinomas were found in 47 female mice (30%) surviving on ENS for 40–65 weeks but none was found in 24 untreated female mice surviving to 77 weeks of age, which is equivalent to 65 weeks in the experimental mice. In addition, liver tumours (adenoma and hepatoma) and pulmonary adenomas and carcinomas were randomly distributed among control and treated animals.

### *Control mice*

The bladder epithelium of the 62 mice (60% of those at start) was essentially normal. Eleven (18%) had evidence of slight focal lymphocytic infiltration, 2 slight chronic inflammation and one a borderline hyperplasia. Twenty bladders were examined radioautographically and [$^3$H]-TdR labelling found to approximate to the normal low value (0·6%) for untreated bladder epithelium (Levi *et al.*, 1969). Occasional labelled cells were found among the non-epithelial cells.

### *ENS for up to* 4 *weeks*

[$^3$H]-TdR incorporation into DNA was estimated biochemically at intervals during the first 4 weeks of ENS treatment (0·005% in diet) (Table II) as a measure of DNA synthesis in the bladder and duodenum. There was an intense stimulation of DNA synthesis in the bladder after 4 days' treatment with ENS in the diet which was of the same order as obtained after stomach tubing. Continued treatment with ENS in the diet led to a progressive lowering in the level of DNA synthesis. Examination of the results from individual mice indicated that in the early period of treatment, [$^3$H]-TdR incorporation was stimulated in the majority of mouse bladders. After 2 and 4 weeks' treatment, DNA synthesis continued to be greatly stimulated in a proportion of animals but had subsided to nearer control levels in the remainder. At 4 weeks the average [$^3$H]-TdR incorporation into male and female bladders was similar. ENS did not affect incorporation of [$^3$H]-TdR into duodenal DNA.

Quantitative assessment of radioautographs of male and female mouse bladders treated for 4 weeks with ENS (Fig. 1; Table III) confirmed the individual variation in response of the bladder at this stage. In general, the intensity of the inflammatory response, the degree of epithelial hyperplasia and the magnitude of the

FIG. 1.—Labelling indices of epithelial and non-epithelial cells of mouse bladder treated with ENS (0·005% in diet) for 4 weeks and given Colchicine (1 mg./kg. s.c.) 5 hours and [$^3$H]-TdR (0·5 $\mu$Ci/g. body weight—i.p.) 1 hour before death.

TABLE I.—*Changes in the Bladder, Liver, Lung and Mammary Gland Induced by ENS* (0·01% *in the Diet*) *in* $A \times IF\ F_1$ *Hybrid Mice*

| | | | | | Bladder | | | | | | Liver | | | | |
|---|---|---|---|---|---|---|---|---|---|---|---|---|---|---|---|
| | | | | | | | | Carcinoma† | | | | | | | |
| Group | Sex | ENS (weeks) | Survival (weeks) | No. of mice | HPI* | Squamous metal plasia | Papilloma† | I | II | Total | Adenoma | Hepatoma† | Lung tumours | Mammary tumours | Other |
| A§ | M | 4 | 4 | 9 | 44 | — | — | — | — | — | — | — | — | — | — |
| B§ | F | 4 | 4 | 9 | 71 | — | — | — | — | — | — | — | — | — | — |
| C | M | 31 | 31 | 17 | 30 | — | — | — | — | — | — | — | — | — | — |
| D | F | 31 | 31 | 25 | 64 | — | — | — | — | — | — | — | — | — | — |
| E | M | 31 | 66 | 25 | 3 | — | — | — | — | — | 1 | 2 | 3 | — | — |
| F | F | 31 | 66 | 27 | 7 | — | — | — | — | — | — | 1 | 7 | — | — |
| G | F | 40–60 | 40–60 | 11 | 36 | 1 | 0 | 2 | 1 | 3 | — | — | 1 | 11 | — |
| H | M | 60–65 | 60–65 | 43 | 32 | — | — | — | — | — | 4 | 3 | 2‡ | — | — |
| I | F | 60–65 | 60–65 | 35 | 36 | 1 | 0 | 2 | 0 | 2 | 1 | — | 2 | 3 | 1 leiomyosarcoma |
| J | M | 62 | 80 | 15 | 9 | — | — | — | — | — | — | — | 3 | — | — |
| K | F | 62 | 80 | 11 | 18 | 2 | 1 | — | — | — | — | — | 4 | 1 | — |
| L | M | 80 | 80 | 19‖ | 39 | 4 | — | — | — | — | — | — | 8 | — | papilloma skin |
| M | F | 80 | 80 | 17‖ | 32 | 1 | — | 1 | 0 | 1 | — | — | 1 | 1 | — |
| N | M | 0 | 65+12 | 38 | 0·5 | — | — | — | — | — | 1 | 11 | 9 | — | 1 lymphosarcoma<br>1 lymphangio-sarcoma |
| O | F | 0 | 65+12 | 24 | 0 | — | — | — | — | — | — | 1 | 2 | — | 1 carcinoma fore-stomach |

* Hyperplastic index (Clayson *et al.*, 1965).

† Most advanced lesion in each mouse.

‡ Also 1 carcinoma bronchus.

§ 0·005% ENS.

‖ 1 mouse killed at 72 weeks when moribund.

TABLE II.—*Effect of ENS on DNA Synthesis in A × IF Mouse Bladder*

| Expt. | Sex | Method of administration of ENS | Time (days) | Control: specific activity DNA c.p.m./μg. deoxyribose | Mean ±s.d. | ENS treated: specific activity DNA c.p.m./μg. deoxyribose | Mean ±s.d. |
|---|---|---|---|---|---|---|---|
| A | F | 0·5 mg. × 3 stomach tube | 4 | 14, 22, 29, 26, 17, 19 | 21 ± 5 | 122, 141, 40, 290, 147, 268 | 168 ± 68 |
| B | F | in diet (0·05%) | 4 | 22, 12, 25, 36, 64, 68, 20, 49, 30, | 41 ± 19 | 77, 183, 175, 285, 563, 171, 293, 357, 407, 63 | 258 ± 146 |
| C | F | in diet (0·05%) | 14 | 22, 37, 32, 31, 37, 20, 40, 43, 41, 60. | 36 ± 11 | 92, 346, 102, 124, 85, 170, 141, 59, 56, 127 | 132 ± 60 |
| D | F | in diet (0·05%) | 28 | 40, 14, 22, 5, 13, 21, 22, 18, 24, 20 | 20 ± 8 | 54, 132, 65, 75, 39, 20, 100, 105, 86, 39 | 69 ± 33 |
| E | M | in diet (0·05%) | 28 | 23, 12, 15, 10, 4 24, 26, 15, 22, 10 | 16 ± 7 | 14, 34, 28, 15, 65, 29, 48, 87, 330, 173 | 82 ± 94 |

TABLE III.—*Effect of ENS (0·005% in Diet) for 4 Weeks on A × IF Mouse Bladder*

| | | | | | Epithelial cells | | | Non-Epithelial cells | | |
|---|---|---|---|---|---|---|---|---|---|---|
| Group | Sex | ENS in diet | Number of mice | Hyper-plastic index | Number of [$^3$H]-TdR labelled cells counted | [$^3$H]-TdR labelling index % | Mitotic index (%) | Number of [$^3$H]-TdR labelled cells counted | [$^3$H]-TdR labelling index % | Degree of inflammation |
| A | M | + | 9 | 44 | 895 | 0·9 (0–3·6)* | 0·07 (0–0·38) | 381 | 1·1 (0·1–5·2) | 0–+ |
| B | F | + | 9 | 71 | 3796 | 5·4 (0·04–17·6) | 0·29 (0–1·03) | 839 | 1·1 (0·4–2·6) | 0–+++ |
| C | M | — | 5 | 0 | 17 | 0·2 (0–0·7) | 0·02† | 77 | 0·28 (0·05–0·6) | 0 |
| D | F | — | 5 | 0 | 6 | 0·06 (0–0·15) | 0‡ | 76 | 0·28 (0·03–0·5) | 0 |

* Range.
† 2 mitoses in 9310 cells.
‡ 0 mitoses in 10,010 cells.

labelling index in both epithelial and non-epithelial cells paralleled each other in individual mice, and was greater in female than in male mice. The female mouse with the highest epithelial labelling index was the only one that showed squamous metaplasia at this stage.

Inflammation was generally lymphocytic in character with some macrophages and plasma cells. In most instances polymorphonuclear leucocytes were also present. The labelling indices of the non-epithelial bladder cells clearly demonstrated that ENS can induce a non-epithelial as well as an epithelial reaction. Although the response of the various types of non-epithelial cells was not assessed quantitatively, labelling was observed to occur in fibroblasts, vascular endothelium and less frequently in muscle cells.

*ENS for* 31 *weeks*

Seventeen male and 25 female mice which had received 0·01% ENS in the diet were killed for histological examination at 31 weeks. There was considerable hyperplasia of the bladder epithelium, expecially in females, but no bladder tumours. The degree of hyperplasia of the bladder epithelium was assessed as the hyperplastic index (Table I) which was 30 for the male and 64 for the female mice. An alternative approach was made possible by the observation (Levi, personal communication) that after 12 weeks' treatment with ENS, the bladder epithelium was populated by small diploid cells whereas the bladder epithelium from untreated mice contains small cells in the basal and intermediate layers and a lumenal layer of large polyploid cells. In mice killed at 31 weeks and later, the bladder epithelium from individual mice has been placed into one of 3 categories: *normal* in which the epithelium was of normal thickness and the lumenal layer consisted of large cells; *hyperplastic* in which only the small cells were apparent; and, *mixed* in which different parts of the epithelium were normal or hyperplastic. At 31 weeks, there were 18% normal and 12% hyperplastic bladder epithelia in male mice, and 12% normal and 40% hyperplastic in female mice.

*ENS for* 40–60 *weeks*

Eleven female mice were killed during this period because of the development of mammary tumours. There was less pronounced hyperplasia of the bladder epithelium than at 4 or 31 weeks, and, in three instances, carcinoma of the urinary bladder (2 Grade I and 1 Grade II)

*Treated mice maintained for* 60–65 *weeks*

Mice which had received ENS for 31 weeks or until death differed mainly in the greater severity of hyperplasia in the epithelium and in the induction of 2 bladder carcinomas (Grade I) in the continuously-treated group. [$^3$H]-TdR labelling in both groups was at a low level similar to that in the controls. Estimation of the epithelial labelling index was not attempted because the infrequency of labelled cells, their random distribution in the epithelium and individual variation between mice meant that little significance would attach to the results unless large numbers of animals were examined.

The hyperplastic index was 32 in continously treated male mice and 36 in female mice. Of the bladder epithelia from these male mice, 37% were normal as judged by the presence of large surface cells and 12% hyperplastic; in the corresponding female mice the values were 43 and 13%, respectively. There were no hyperplastic epithelia in either male or female mice treated with ENS for 31 weeks and killed at 66 weeks.

*Mice killed at* 80 *weeks*

Some of these mice had been maintained continuously on ENS whereas others were returned to the basal diet at 62 weeks (Table I). Although there are no strict controls, the infrequency of hyperplasia or naturally occurring tumours in the murine bladder epithelium justifies the conclusion that changes in the bladder epithelium of these mice may be regarded as chemically induced.

The severity of hyperplasia found in continuously treated female mice was, at 80 weeks, slightly less than that found at 65 weeks and considerably less than at 4 or 31 weeks, whereas the level of hyperplasia in the male mice was essentially unchanged throughout the experiment. The hyperplastic index was 39 in male and 32 in female mice. There were 42 and 35% normal epithelia as judged by the presence of large surface cells in male and female mice, respectively, and 21 and 6% were hyperplastic.

Bladder epithelium from three mice was of mixed type and there was squamous metaplasia in two, of 11 female mice which had received the basal diet for 18 weeks before death. One mouse had a malignant papilloma. Epithelium of mixed type was present in five of 15 similarly treated male mice. Squamous metaplasia was present in five and bladder carcinoma in one of the continuously treated female mice. These facts suggest that the changes in the bladder epithelium at this time may not be completely dependent on the continued presence of ENS.

### DISCUSSION

The number of bladder tumours induced by ENS in A × IF mice was lower than previously found in either Ab × IF or C57 × IF mice (Clayson *et al.*, 1967). It is possible that the A × IF mice are intrinsically less sensitive to ENS but, alternatively, the low incidence of bladder tumours may be a result of the condition of many of the mice during the experiment. This opinion is reinforced by the fact that the Ab × IF mice used by Clayson and Bonser (1965) developed a higher incidence of bladder tumours despite the Ab parents being derived by Caeserian section from an A mouse of the line used in this experiment.

The finding of a 30% incidence of mammary carcinomas with ENS is the first association of tumour formation outside the urinary tract with this chemical. It is difficult to assess the part played by ENS in the development of these tumours. The A strain parent carries the mammary tumour virus and, according to Streeter (1960), had a 4% incidence of mammary carcinomas in virgin females and an 80% incidence in breeding females. The female IF mammary gland, on the other hand, is mammary tumour virus free but is susceptible to tumour induction by certain chemical carcinogens. Furthermore, although untreated A × IF female mice, and those treated with ENS for only 32 weeks, in the present experiment, did not develop breast tumours, some have been observed in other untreated mice of this hybrid. It is not possible to decide whether the mammary tumours reported here are a result of the direct carcinogenic action of ENS or of a synergism with other factors such as the mammary tumour virus.

ENS-induced urinary tract hyperplasia requires the continued presence of the chemical for its maintenance at 1 (Sen Gupta, 1962) and 31 weeks after the start of treatment and to a lesser extent at 62 weeks. The failure of the hyperplasia in the bladder epithelium to regress completely between 62 and 80 weeks of treatment may indicate that it is no longer dependent on the continued presence of the chemical or may be because the relatively long life span of the epithelial cells leads to their retention by the epithelium for a considerable time after the removal of the stimulus. The longevity of the epithelial cells is indicated by the small number of cells incorporating [$^3$H]-TdR after 60–65 weeks' treatment with ENS.

The acute proliferative response in the bladder epithelium following the start of ENS treatment diminishes in the first 4 weeks in many of the mice. At this time there is an appreciable response to the chemical in the non-epithelial cells in some mice. Epithelial hyperplasia, which develops within a few days after the start of ENS treatment (Sen Gupta, 1962; Levi *et al.*, 1969) appeared to be maintained at a high level for at least 30 weeks but was less severe by 60 or 80 weeks in the female mice. In the male mice, however, a lower mean level of hyperplasia was maintained throughout the experiment. These results are capable of explanation in one of two ways: either the cells in the bladder epithelium lose their sensitivity to ENS or its active metabolite, or the metabolism of the drug changes during continuous treatment and the active material no longer reaches the bladder epithelium in sufficient amounts to stimulate the cells to division.

### SUMMARY

1. 4-Ethylsulphonylnaphthalene-1-sulphonamide (ENS) induced bladder tumours in female A × IF $F_1$ hybrid mice. The incidence of bladder tumours was lower than has been recorded with other hybrid mice.

2. Measurement of tritiated thymidine incorporation into the bladder of ENS-treated mice demonstrated that the acute proliferative response to ENS subsided in a large proportion of the mice during the first 4 weeks of treatment.

3. Radioautography of bladders from mice treated with ENS for 4 weeks confirmed that the epithelial proliferation was less than in mice given a single oral dose of the drug. By 65 weeks there was little difference between bladder epithelium from treated and control mice.

4. ENS-induced epithelial hyperplasia was less marked in female mice after 65 and 80 weeks of treatment than after 4 and 31 weeks. The large surface cells were re-established in the whole or part of the bladder epithelium of more than three quarters of these mice in the later stages of the experiment. Hyperplastic changes in male mice were less prominent than in female mice at 4 and 31 weeks.

5. After 4 weeks of treatment, there was a small but appreciable response to ENS in the non-epithelial cells of the bladder of some mice involving fibroblasts, the lining cells of the blood vessels and, less frequently, muscle cells.

We thank Professor E. H. Cooper for his interest and the Yorkshire Council of the British Empire Cancer Campaign for Research for financial support. O.C. was in receipt of a scholarship from the British Council and F.K.D. was the holder of a Training Fellowship from the International Agency for Research on Cancer, Lyon, France.

### REFERENCES

BRIMELOW, H. C. AND VASEY, C. H.—(1958) U.K. Patent No. 791, 529.

CLAYSON, D. B. AND BONSER, G. M.—(1965) *Br. J. Cancer*. **19**, 311.

CLAYSON, D. B., LAWSON, T. A., SANTANA, S. AND BONSER, G. M.—(1965) *Br. J. Cancer*, **19**, 297.

CLAYSON, D. B., PRINGLE, J. A. S., AND BONSER, G. M.—(1967) *Biochem. Pharmac.*, **16**, 619.

LAWSON, T. A., DZHIOEV, F. K., LEWIS, F. A. AND CLAYSON, D. B.—(1968) *Biochem. J.*, **111**, 12P.

LEVI, P., COWEN, D. M. AND COOPER, E. H.—(1969) *Cell and Tissue Kinetics*, **2**, 249.

PAGET, G. E.—(1958) In ' A symposium on the Evaluation of Drug Toxicity ', edited by A. L. Walpole and A. Spinks. London (Churchill).

SEN GUPTA, K. P.—(1962) *Br. J. Cancer*, **16**, 110.

STREETER, D. J.—(1960) " A study of ovarian changes in three strains of mice and their hybrids following skin application of a chemical carcinogen". M.Sc. Thesis: University of Leeds.